# The Multiple Silicone Tube Device, “Tubes within a Tube,” for Multiplication in Nerve Reconstruction

**DOI:** 10.1155/2014/689127

**Published:** 2014-04-17

**Authors:** Fredrik Johansson, Lars B. Dahlin

**Affiliations:** ^1^Department of Biology/Functional Zoology, Lund University, Sölvegatan 35, 223 62 Lund, Sweden; ^2^Department of Clinical Sciences in Malmö/Hand Surgery, Lund University and Skåne University Hospital, Jan Waldenströms Gata 5, 205 02 Malmö, Sweden

## Abstract

Multiple nerve branches were created during the regeneration procedure after a nerve injury and such multiple branches are suggested to be used to control, for example, prosthesis with many degrees of freedom. Transected rat sciatic nerve stumps were inserted into a nine mm long silicone tube, which contained four, five mm long, smaller tubes, thus leaving a five mm gap for regenerating nerve fibers. Six weeks later, several new nerve structures were formed not only in the four smaller tubes, but also in the spaces in-between. The 7–9 new continuous nerve structures, which were isolated as individual free nerves after removal of the tubes, were delineated by a perineurium and contained both myelinated and unmyelinated nerve fibers as well as blood vessels. Stimulation of the proximal nerve elicited contractions in distal muscles. Thin metal electrodes, inserted initially into the smaller tubes in some experiments, became embedded in the new nerve structures and when stimulated contractions of the distal muscles were observed. The “tubes within a tube” technique, creating multiple new nerves from a single “mother” nerve, can be used to record multiple signals for prosthetic device control or as sources for supply of multiple denervated targets.

## 1. Introduction

The clinical treatment of nerve injuries is complex and the outcome depends on a variety of factors; one of them is performing an optimal reconstruction of the severed nerve trunk. In some of these situations, multiplication of the proximal nerve trunk would be advantageous, like in encountering extensive traumatic brachial plexus injuries and/or when multiple targets, supplied by a single nerve, are denervated, as well as when multiple signals for prosthetic arm/hand device control are required.

An artificial limb, in particular arm/hand prosthesis, must be endowed with several motors for the control of the movements of individual fingers, the wrist, the elbow, and the shoulder [1]. Today, there are robot hands existing with 16–24 degrees of freedom of movement (DOF) [2–7], which are values close to the 21 DOF of the human hand [2]. It is difficult to obtain a sufficient number of nerve signals to run such a complicated prosthesis. Moreover, there are around 17000 receptors in a human hand, so if sensory feedback is considered, the number of required contact points increases dramatically. A solution for motor control of an advanced prosthesis could be implantable electrodes with high spatial resolution, which via telemetry could be used to control the artificial limb. Electrode arrays with up to 100 electrodes have been used for recordings in peripheral nerves [8]. External electrodes, picking up myoelectrical (EMG) signals, can also be utilized, but again the numbers of signals are limited. There are also problems with the attachments of the electrodes to the skin. Still, it has been demonstrated that EMG signals can be used to control a hand prosthesis with several degrees of freedom [9, 10].

For invasive electrodes, one possibility would be to increase the number of fascicles in a severed nerve. Assume that a nerve can be divided into two, three, or more parts. Then, one would be able to selectively address two, three, or more individual nerve trunks or even fascicles. We hypothesize that splitting of a nerve into multiple parts can offer a solution to increase the number of signals required for an advanced arm/hand prosthesis. Interestingly, it has been demonstrated that a severed nerve can be redirected to several new muscles, thus creating a platform for EMG control of a prosthetic device [11–13]. This technique is referred to as targeted reinnervation [14] and it can also be used for sensory feedback.

If the continuity of a peripheral nerve is disrupted, then it too can be repaired by joining the severed ends via a tube [15–18]. A new nerve trunk forms in the tube and nerve fibers grow from the proximal to the distal nerve within a few weeks depending on the distance between the nerve ends.

In the present study, we tested if smaller tubes, inserted within a larger tube, could be used to create multiple new nerves or “fascicles,” thus offering the possibility to obtain multiple signals from one and the same nerve. Such a “Matruschka” or Russian doll approach could be used for bidirectional interfacing of a nerve or as a supply for multiple denervated end organs as in traumatic brachial plexus injuries. To test this possibility, we repaired the rat sciatic nerve by one large tube containing four smaller ones. This setting resulted in the formation of up to nine new fascicles (i.e., minor nerve trunks), each of which was surrounded by a perineurial-like structure. We could also show that these new nerves could be stimulated to induce muscle contraction distal to the site of nerve reconstruction.

## 2. Materials and Methods

### 2.1. Tubes with Multitubes

**Figure 1:**
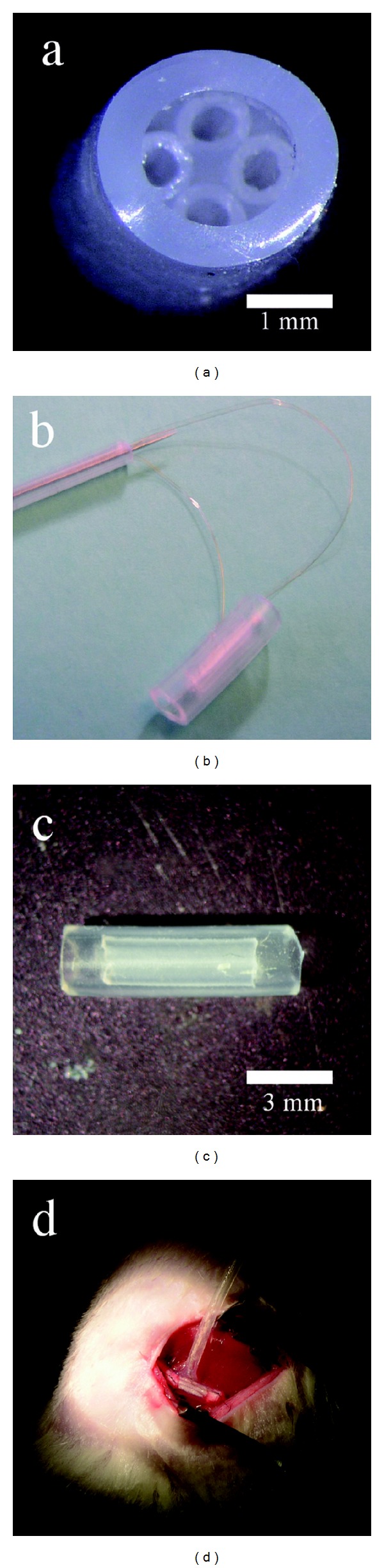
Four, five mm long, small diameter tubes were inserted into a nine mm long large diameter silicon tube ((a) and (b)). Two constructs were made: one without ((a) and (c)) and one with electrodes ((b) and (d)). In the latter case, a long silicone catheter was attached to the “tubes within a tube” chamber resulting in T-shaped chamber. The long tube housed the approximately 4 cm long Pt/Ir electrodes, which ended inside two of the smaller tubes.  Figure 1(c) shows the catheter with the electrode wires before it is glued to the “tubes within a tube” chamber.  Figure 1(d) shows the T-tube construct in situ. The long catheter containing the electrode leads was positioned in a pocket made subcutaneously before the wounds were closed by Agraff clips.

Two variations of silicone tubes were used: one without (Figures 1(a) and 1(c)) and one with electrodes (Figures 1(b) and 1(d)). In the former construct, the outer tube was nine mm long and with an outer diameter of three mm and inner diameter of two mm. In this tube, four, five mm long, tubes, with an outer diameter of 0.8 mm and inner diameter of 0.5 mm, were inserted. For nerves receiving electrodes, the same basic design was used, but now a longer tube was attached to the outer chamber in a T-like manner to host the electrode connections (Figures 1(b) and 1(d)). In this paradigm, two of the four inner tubes were endowed with 30 *μ*m thick (5 *μ*m isolation, 25 *μ*m conductor) Pt (90)/Ir (10) electrodes (Goodfellow, England). The isolation was removed from 3 mm of the electrode within the small tubes.

### 2.2. Animals and Surgical Procedures

Female Sprague-Dawley rats, weighing around 200 g, were used. The Ethical Committee at Lund University, Sweden, approved the study. A total of 10 rats were used. Four of these received implanted electrodes. The animals were anesthetized using a mixture of diazepam and pentobarbital as described [19] and the sciatic nerve was exposed in the thigh region. The nerve was transected and the above-described tubes were used to join the severed ends of the sciatic nerve. The nerve stumps were inserted two mm into the larger tube and secured with single sutures. The wounds were closed and the animals were allowed to recover for six weeks.

### 2.3. Evaluations

#### 2.3.1. Electrophysiology

**Figure 2:**
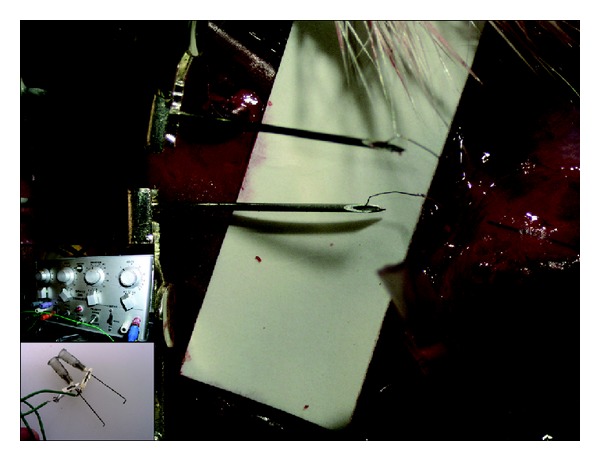
*Large picture*: needles (0.4 mm) contacting electrode leads for stimulation via the Pt/Ir electrodes in the smaller tubes.* Upper insert*: stimulator delivering rectangular pulses of 1 ms duration at 10 Hz and different amplitudes (0.1–1 V).* Lower insert*: electrodes needles (0.7 mm) for stimulation of the sciatic nerve proximal to the tube repair site.

At the day of evaluation, the animals were reanesthetized and the tube, with the attached sciatic nerve, was exposed. Needle electrodes were used for stimulation, providing square pulses of 10 Hz, 1 ms duration with an amplitude of 0.2 V to 1 V. The stimulation was delivered either proximal to the chamber or via the leads to the internal electrodes of the smaller tubes (Figure 2). Visual observation of flexion of the foot or contraction distal to the site of repair at a frequency of the stimulation, that is, 10 Hz, would signify that axons had regenerated through the tubes into the distal muscles. In some experiments, the undamaged contralateral sciatic nerves were exposed to determine the threshold for muscle contraction.

#### 2.3.2. Morphology

Following stimulation, the nerve and the tube were removed and placed in Stefanini's fixative. The tubes were longitudinally sliced to facilitate penetration of the fixative. After overnight fixation the preparations were washed, cryoprotected in PBS, containing 20% sucrose, and sectioned in a cryostat. The sections (10 *μ*m) were either processed for conventional histology, using toluidine blue, or stained by immunocytochemistry for neurofilaments to visualize axons. The primary antibody (dilution 1/200) used was a rabbit anti-Neurofilament M (145 kD; polyclonal; Chemicon/Millipore, Germany), which has a high specificity, followed by a goat anti-rabbit Alexa 488 antibody (Molecular probes, USA). Counterstaining with bisbenzimide was used to visualize nuclei in the sections. The procedure has been described in detail elsewhere [19].

## 3. Results

### 3.1. Electrophysiology

Four individual rats were stimulated proximal to the site of tube repair. All rats showed contraction of muscles distal to the tube. Stimulation through the electrodes embedded in the chamber with an elicited muscle response was successful in three out of four cases. A stimulation amplitude of 0.3 V and 1 ms duration was sufficient to cause contractions (film sequence reference). Stimulation of the contralateral uninjured sciatic nerve required between 0.2 V and 0.25 V at 1 ms duration to induce muscle contraction visible by the naked eye.

### 3.2. Morphology

**Figure 3:**
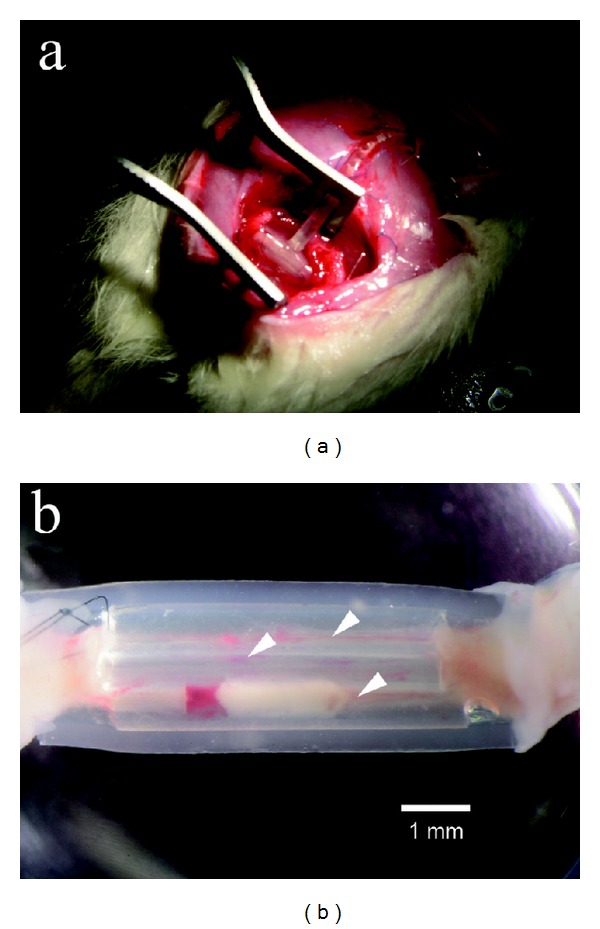
(a) Macroscopic image of a tube at six weeks in situ. (b) Tube construct removed from the animal. Nerve structures with blood vessels are visible within three of the smaller tubes (arrowheads).

**Figure 4:**
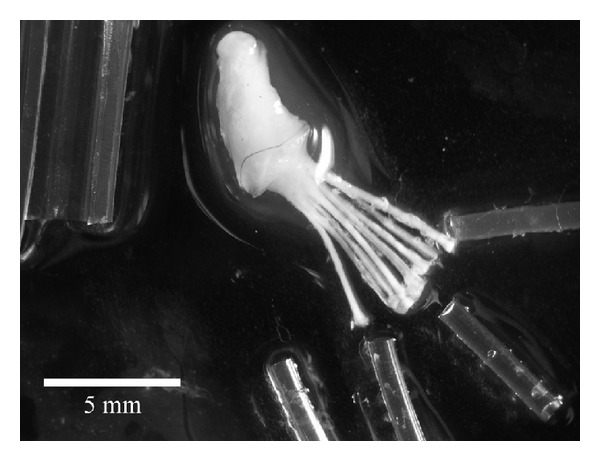
Dissected preparation. The large and smaller tubes have been removed and the new nerve structures lay bare. Eight new nerve structures are visible.

**Figure 5:**
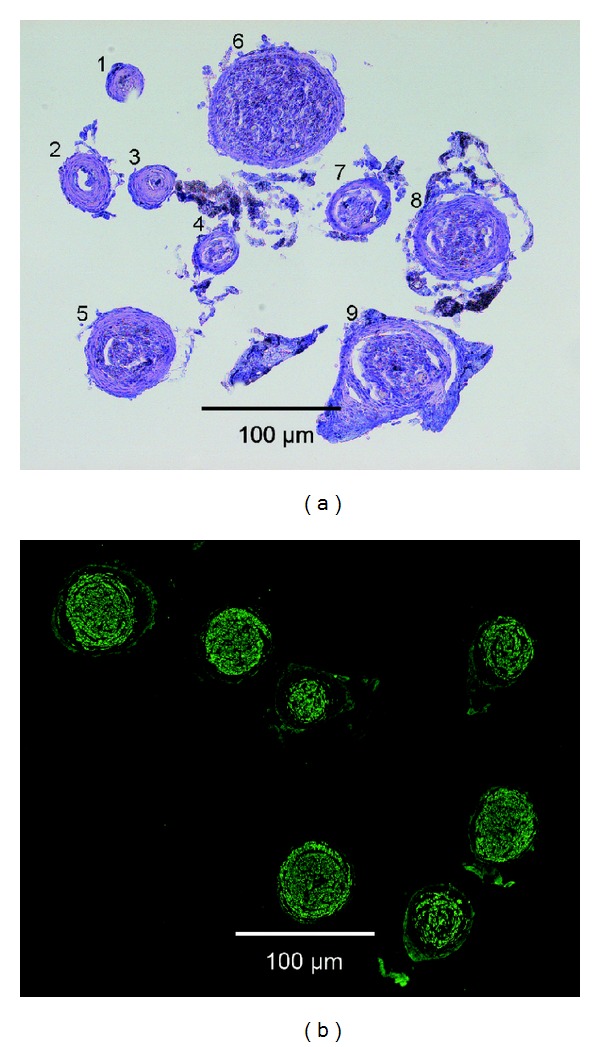
Cross-section of the new nerve structures. (a) Preparation stained with toluidine blue. Nine new nerve structures are visible, some containing many fascicles/axons (e.g., numbers 5, 6, 8, and 9), while some (e.g., numbers 2 and 3) fascicles contain mostly connective tissue with only a few fascicles and hence axons. (b) Neurofilament staining reveals that axons are present in the new nerve structures. This particular preparation contained seven new nerves.

**Figure 6:**
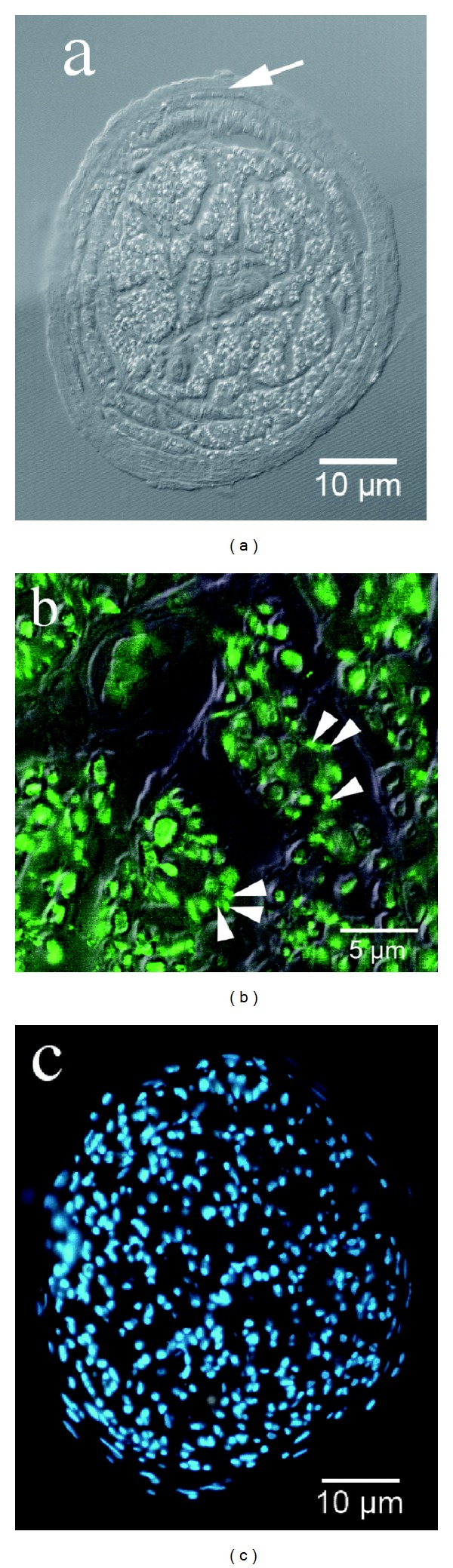
(a) The new nerve structure is enclosed by a perineurium (arrow) and both blood vessels as well as unmyelinated (arrow heads; b) and myelinated axons are visible (combined Normarski and fluorescence microscopy). (b) Neurofilament staining: both myelinated and unmyelinated axons (arrow heads) appear green fluorescent. (c) Nuclear staining of the new nerve structure.

**Figure 7:**
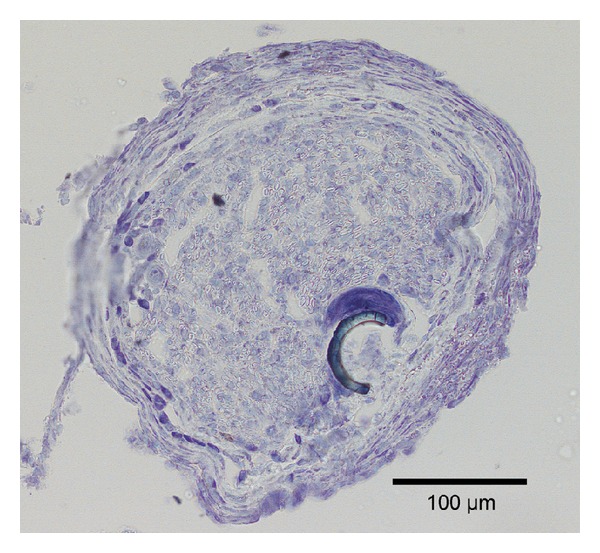
Section of new nerve structure with electrode residue. Note the large cell(s), probably an inflammatory cell, attached to the electrode residue.

Structures had been formed within the tubes joining the two cut ends of the sciatic nerve (Figure 3). Removal of the outer and inner tubes showed that there were a total of seven to nine new small nerve trunks in the tube within tube chamber (Figures 4 and 5). After sectioning, the new nerves could be studied in the light microscope. The nerve structure was either round or irregular depending on if they were formed within a tube or formed within an area between the tubes. A perineurium-like sheath surrounded the small nerve trunks (Figure 6(a)). In the endoneurium, blood vessels and myelinated axons were readily observable. Immunostaining for neurofilaments showed that there were also unmyelinated fibers in the endoneurium of the individual new small nerve trunks (Figure 6(b)). The counterstaining with bisbenzimide for nuclei identification revealed an apparent normal distribution of perineurial nuclei, Schwann cell nuclei, and endothelial cell nuclei in the blood vessels (Figure 6(c)). There was an empty space left by the electrode, which was surrounded by mononuclear inflammatory cells (Figure 7).

## 4. Discussion

Tube repair of nerves is not a new idea [for review see [20–24]] and already in the early 20th century bone was used to bridge nerve defects. Later, blood vessels were used, followed by preformed mesothelial chambers, silicone tubes, and a variety of biodegradable tubes or “tubes” made of rolls of placental membranes and teased tendons. The concept works also in humans [22, 25, 26] Actually, a conduit, or even a guiding structure like longitudinal sutures [15], in-between a proximal and distal stump seems to be the only requirement for a nerve to reform providing the distance between the stumps is not too long, unless a stepping stone procedure is not created [16]. The new nerve is formed by the migration of Schwann cells, fibroblasts, and new blood vessels and outgrowth of axons into a matrix of fibrin, which are the basic requirements for regeneration.

The regenerative capability of the peripheral nervous system is truly remarkable. Even in mammals, it is sufficient to supply a piece of a nerve or muscle distally in the tube to induce formation of a nerve trunk. Furthermore, even if two degenerating pieces of a nerve are attached to both ends of a tube, a new nerve-like structure is formed in the tube; the nerve-like structure is being used as a nerve graft [27]. Here, we found that multiple new nerves could be produced from a single “mother” nerve if the proximal stump was presented to several small tubes within a larger tube. Conventional histology and immunohistochemistry revealed the formation of new nerve structures with an overall structure and with a composition similar to those observed after repair of an injured nerve with tubes, that is, a normal nerve with myelinated and unmyelinated nerve fibers and blood vessels surrounded by a perineurium. Furthermore, it was possible to stimulate the new nerve structures at thresholds similar to that observed in the intact sciatic nerve and we observed reinnervation of the targets, that is, muscles, distal to the tube repair. In our paradigm, there were four tubes and five empty spaces in-between (Figure 1(a)). Indeed, we found that the sciatic nerve, with a typical diameter of approximately 1.2 mm, had the ability to split into at least nine new fascicles or nerves. There is no reason to assume that we have reached any limit and larger nerves, like those in the human arm or leg, can probably be split into many more fascicles. This would allow for an interface controlling the most advanced upper limb prosthetic devices available, which requires the control of 21 motors. Furthermore, each small tube could be treated with either skin or muscle homogenate. This neurotrophic influence can induce separation of sensory and motor fibers into different tubes [28, 29], resulting in the possibility of addressing both types of fibers separately, thus, for at true bidirectional interface/prosthesis. The probability of keeping the native fascicle organization after splitting the nerve into many smaller ones is rather low as in all nerve regeneration therapies after a nerve transection. Such mismatch is to a large degree compensated by the remarkable plasticity of the brain, which is also observed after different nerve transfers clinically. Hence, the cerebral plasticity may enable functional outcome after such procedures to some extent, particularly for motor function. In the case of a computer-assisted prosthesis, artificial neuronal networks may do a similar task for controlling the device, for example, using some principal component analysis of the recorded nerve signals. To determine the exact origin of the specific axons in each new fascicle, one may consider using retrograde tracing by applying different dyes to the appropriate fascicles. If traumatic brachial plexus injuries are considered, there should, with the present paradigm, be no shortage of fascicles for reinnervation of multiple targets. An exciting possibility is to test if the present paradigm could be useful for reinnervation of targets below a spinal cord injury.

The finding that the new nerve structures were surrounded by a perineurial-like structure is an advantage. The perineurium offers isolation and should prevent overhearing between electrodes as compared to an array of multiple electrodes inserted into one and the same fascicle. In another technique, where longitudinal sutures are used as guidance, a perineurial-like structure is also formed, but it encloses the whole new nerve structure [16]. Another advantage is that we used longitudinal electrodes, which increase the surface area for signal stimulation and signal pickup as compared to sieve electrodes. Taken together the present technique with tubes within a tube should offer a new avenue for nerve repair in various nerve repair settings, including those involving prosthetic devices.
